# Design, development, and evaluation of a surveillance system for suicidal behaviors in Iran

**DOI:** 10.1186/s12911-022-01925-3

**Published:** 2022-07-11

**Authors:** Mohsen Shafiee, Mohammad Mahboubi, Mostafa Shanbehzadeh, Hadi Kazemi-Arpanahi

**Affiliations:** 1 Department of Nursing, Abadan University of Medical Sciences, Abadan, Iran; 2 Department of Public Health, Abadan University of Medical Sciences, Abadan, Iran; 3 Department of Health Information Technology, School of Paramedical, Ilam University of Medical Sciences, Ilam, Iran; 4 Department of Health Information Technology, Abadan University of Medical Sciences, Abadan, Iran; 5 Department of Student Research Committee, Abadan University of Medical Sciences, Abadan, Iran

**Keywords:** Suicide, Suicide behavior, Surveillance system, Minimum data set

## Abstract

**Background:**

Suicide is a serious cause of morbidity and mortality in Iran and worldwide. Although several organizations gather information on suicide and suicide attempts, there is substantial misperception regarding the description of the phenomenon. This study proposes the minimum data set (MDS) for suicidal behaviors surveillance.

**Methods:**

A literature review was first conducted to achieve a thorough overview of suicide-related items and map the existing evidence supporting the development of the MDS. The data items included in the literature review were then analyzed using a two-round Delphi technique with content validation by an expert panel. The suicidal behaviors surveillance system was then established based on the confirmed MDS, and ultimately, its performance was assessed by involving the end-users.

**Results:**

The panel of experts consisted of 50 experts who participated in the Delphi phase and validity content review. Of these, 46% were men, and their mean age and average work experience were (36.4, SD ± 6.4) and (12.32, SD ± 5.2) years, respectively. The final MDS platform of our study contained 108 items classified into eight main categories. A web-based system with a modular and layered architecture was developed based on the derived MDS.

**Conclusion:**

The developed system provides a framework for recording suicidal behaviors' data. The integration of multiple suicide-related information systems at the regional and national levels makes it possible to assess the long-term outcomes and evolutions of suicide prevention interventions.

## Background 

Suicide is a serious unprecedented social health crisis [1]. It is the cumulative result of socio-economic, cultural, environmental, biological, psychological, and clinical factors [2]. The path to suicide is complex, including a continuum from ideation, planning, attempt, and completion. For everyone who dies by suicide, nearly 20 to 30 suicide attempts are made, and the history of unsuccessful suicide attempts remains a crucial predictor of complete suicide. Thus, in addition to suicide deaths, suicidal ideations and nonfatal suicide attempts have also captured the attention of academics, researchers, health authorities, and politicians for suicide prevention [3–5]. According to the World Health Organization (WHO), globally, more than one million individuals terminate their lives by suicide annually. Suicide is the second cause of death in people aged 15 to 29 and the third cause of death for individuals aged 15 to 44 years [6, 7]. This painful phenomenon is defined as a planned deadly self-harm behavior intending to kill oneself [8]. This serious health problem is dramatically deteriorating, as the age-adjusted amount of suicides and nonfatal self-inflicted injuries has increased by 33% and 40%, respectively [9]. In Iran, suicide mortalities increase with an expected average rate of 9.9 per 100,000 individuals annually [10]. Still, these statistics are the tip of the iceberg, and population-specific statistics are not widely available. One widespread belief among investigators is that suicides are generally underreported**,** and some investigators believe that suicide tolls are miscalculated by 20–25% or higher [11]. This representation of suicide does not correspond to the facts about this phenomenon and obscures significant variability in suicide rates and patterns among districts and communities. Suicide underreporting may mislead public health authorities [12]. For these reasons, the necessity for amended and extended surveillance of suicide to reinforce the evidence base for prevention is well identified. The WHO recommends that all countries must design and build suicide prevention systems in accordance with their norms and technical infrastructures [13]. Therefore, a major goal of the global public health is preventing and recording suicide information.

A surveillance system is a useful way to record suicide information. In general, the lack of a national suicide prevention strategies in Iran, lack of reliable data for suicide surveillance, incomplete reporting, incorrect classification of suicidal deaths due to religious, social, and cultural stigma, as well as the lack of an effective recording system are the main challenges to address in this regard [14, 15].

Given the gross under-reporting of suicide and attempted suicide in Iran, it is important to establish an inclusive surveillance system to collect suicidal behaviors data to more accurately evaluate the efficiency of suicide prevention programs. Generally, suicide recording is an intricate, multilevel process consisting of medical and legal matters, including accountable authorities that differ across countries [11, 16]. The lack of a systematic suicide surveillance system leaves a large gap in our understanding of suicide behavior cases. It also hinders our efforts to precisely screen suicide occurrence, demographic patterns, and time-based variations of suicide methods, and thus impacts the development and assessment of suicide anticipation plans at different levels [17]. Determining the topographical and regional dispersion of suicidal predictors can inform health policy-makers in developing more well-organized suicide prevention interventions. These contributing factors are specific to each country and are essential to completely recognize these behaviors.

At present, there is no national surveillance system for suicidal behaviors in Iran. A consistent data set is required to help represent and characterize such incidents, describe contributing factors and conditions, and provide information for prevention at the local, provincial, and national levels [18, 19].

Building an information system according to the system development life cycle (SDLC) methodology will promote its integrity and enterprise practicality. Data consistency and coordination is the first significant phase in the information system SDLC and should be achieved in compliance with an appropriate plan [20, 21]. Therefore, in the initial phase of our study, the minimum data set (MDS) of suicidal behaviors was developed to determine system data requirements. MDS is a standardized data collection method and provides precise access to clinical data. To build the suicidal behaviors surveillance system, MDS enables enhanced progress in standardized gathering, understanding, judgment, and combination of data.

The current study aimed to design, develop, and evaluate a surveillance system for suicidal behaviors. To this end, a comprehensive MDS was first developed as a framework for designing a suicidal behaviors surveillance system; then, the system was developed and its usability was evaluated based on its users’ standpoints.

## Methods

### **Study design**

To design a suicidal behaviors surveillance system, five stages were followed: (1) A literature search to extract potential data items related to suicidal behaviors, (2) Conducting a two-round Delphi survey to rank data items, (3) Content validity evaluation of data items, (4) Developing the surveillance system, and (5) Conducting a survey to evaluate the surveillance system based on users’ opinions.

#### Literature review

**Fig. 1:**
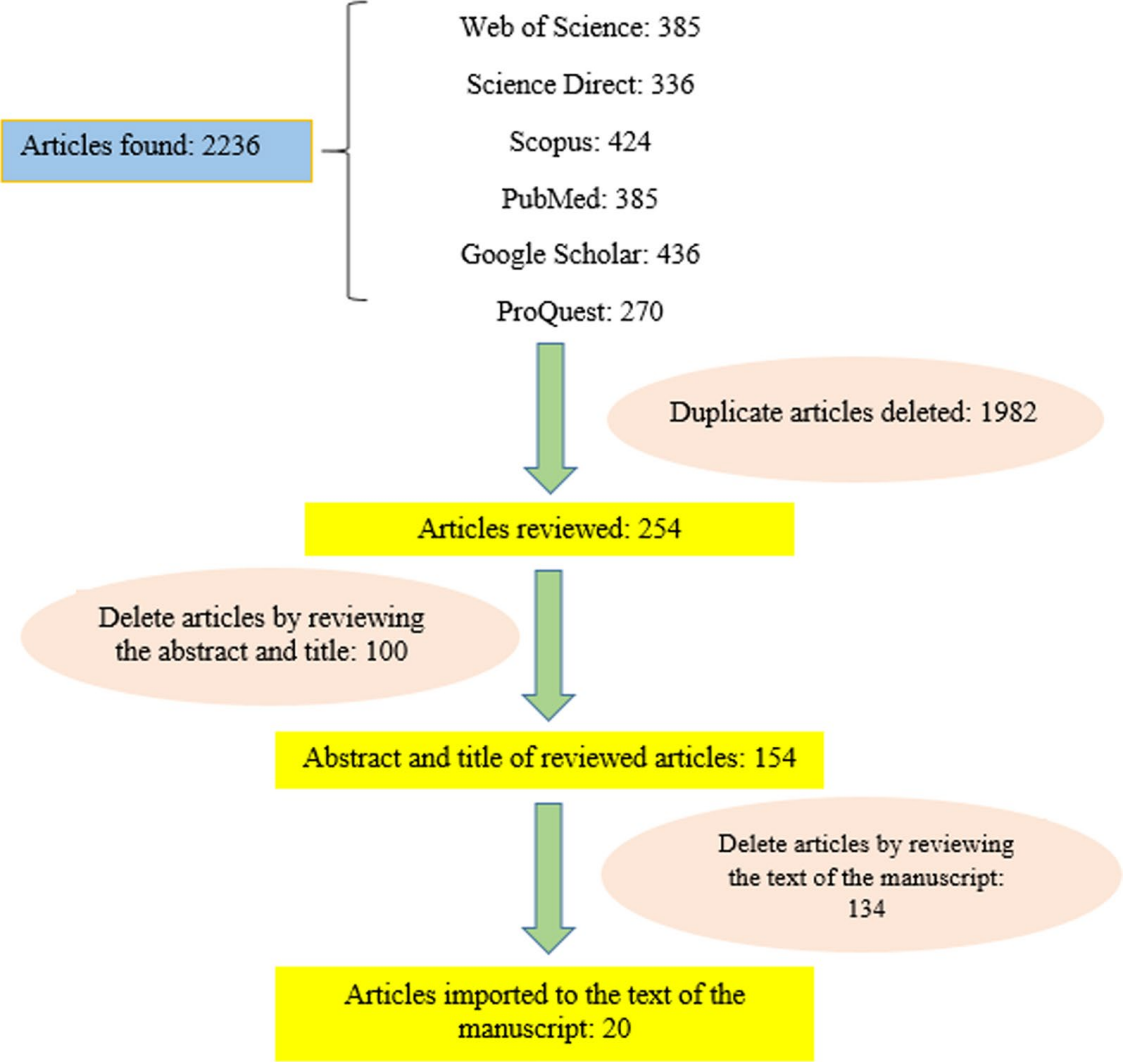
Search flow diagram

To retrieve relevant resources, scientific databases such as the Web of Science, PubMed, Scopus, Google Scholar, SID, and MagIran were reviewed. The inclusion criteria were full-text journal articles in Persian and English published from 2000 to 2022. Letters to the editor, editorials, news, meeting abstracts, short communications, conference papers, and journal articles with no full-text available were excluded (Fig. 1). Any research that studied risk factors, circumstances, nature, population subgroups, and any other aspect of suicide and attempted suicide was included (Table 1).

**Table 1:** search strategy

Database	Search syntax
Scopus	(TITLE-ABS-KEY ("Registry system") OR TITLE-ABS-KEY ("Surveillance system”) OR TITLE-ABS-KEY ("Information system”) OR TITLE-ABS-KEY ("Data management”) OR TITLE-ABS-KEY ("Data system “) OR TITLE-ABS-KEY ("Information management “) AND TITLE-ABS-KEY (Suicide) OR TITLE-ABS-KEY (“Self-harm”) OR TITLE-ABS-KEY (“Self-injury”) OR TITLE-ABS-KEY (“Self-mutilation”) AND (LIMIT-TO (LANGUAGE, "English”)) AND (LIMIT-TO (2010- 2021))
Science Direct	TITLE-ABS-KEY ("Registry system “OR “Surveillance system “OR “Information system “OR “Data management “OR “Data system” OR “Information management ") and TITLE-ABS-KEY (Suicide OR Self-harm OR Self-injury OR Self-mutilation) AND English [Lang], limited to 2010- 2021
Web of Science	(TC = (" Registry system “OR” Surveillance system “OR” Information system “OR” Data management “OR” Data system" OR “Information management”) AND TC = (Suicide OR Self-harm OR Self-injury OR Self-*mutilation*) AND LANGUAGE: (English), limited to 2010- 2021
PubMed	(((((((((((((("Registry system "[Title/ Abstract]) OR " Surveillance system "[ Title/ Abstract]) OR " Information system "[ Title/ Abstract]) OR " Data management "[ Title/ Abstract]) OR " Data system "[ Title/ Abstract]) OR " Information management "[ Title/ Abstract]) AND Suicide [Title/ Abstract]) OR Self-harm [Title/ Abstract]) OR Self-injury [Title/ Abstract]) OR Self-mutilation [Title/ Abstract]))) AND (English[lang]), limited to 2010- 2021
Google Scholar	allintitle: " Registry system " OR " Surveillance system " OR “Information system” OR “Data management” OR “Data system” OR “Information management” AND " Suicide" OR " Self-harm" OR " Self-injury" OR " Self-mutilation" AND English[lang], limited to 2000- 2021

Extracted items related to suicidal behaviors were entered into a checklist with two administrative and clinical sections. The checklist included items related to suicide behaviors extracted from the literature review. We needed this checklist to conduct the Delphi study and build an initial platform of MDS. In the second step, the medical records of suicide patients at Ayatollah Taleghani and Shahid Beheshti hospitals affiliated with Abadan University of Medical Sciences (Iran) were reviewed, and relevant data were also entered into the checklist.

#### Delphi survey

Providing a valid MDS is the first step to developing surveillance systems. To design the MDS, an electronic checklist was prepared from the extracted factors related to suicidal behaviors. In the Delphi survey, there is no specific way for selecting the participants, but they can be selected according to homogeneity, study time, extension range, availability, and objectives of the study [22, 23]. Herein, a consistent sample of experts who dealt with individuals who have suicidal behaviors was selected. In Delphi surveys, when the panel of experts is homogeneous, the suggested sample size ranges from 10 to 15 in various studies; however, in our study, 50 people based on the available experts were selected to reduce the error rate [24, 25]. To determine the final suicide behaviors surveillance MDS, the expert panel was chosen according to the following criteria: (1) The participants had to be selected from disciplines related to suicide; (2) Experts in any field had to have more than three years of work experience, have a related academic degree, and if possible, have related scientific publications and professional working experience; (3) Participants have to return their responses to the researchers (if a questionnaire was not returned, the participant would be excluded).

The preliminary data items were used as the survey to elicit panelists’ views regarding the core data items of suicidal behaviors surveillance MDS. For inclusion in the electronic checklist of the suicidal behaviors surveillance system, each data item was ranked through a two-round Delphi technique. The experts who joined the survey were asked to allocate an importance value to each data item on a five-point Likert scale, ranging from 1 representing the “lowest level of importance” to 5 representing the “highest level of importance”. The participants were blind to one another's responses throughout the survey. They were also asked to suggest new items that were not registered in the preliminary data set for subsequent ranking. The content validity of the questionnaire was evaluated by an expert panel, including instrument developing experts (two), psychologists (two), health information managers (two), psychotherapists (two), and epidemiologists (two). The test–retest method was adopted to assess the reliability of the questionnaire. The item would be included in the final MDS if it achieved an agreement of ≥ 75% (e.g., of an item’s importance).

A two-stage Delphi survey was conducted to determine the important items that meet the criteria for inclusion in the suicidal behaviors surveillance MDS. A panel of experts, including 50 people, was formed. First, the subject and purpose of the study were sent to the experts through letters and emails, and informed consent for participation was received from them. Then, the electronic questionnaire was emailed to them. A period of two weeks was considered to fill out the questionnaire. To reduce the error, a team of experts blindly evaluated the scores of these items. The evaluation in the Delphi stage is as follows: if < 60% of the experts agree with the importance of an item, that item will be removed. If 60–75% agree with the importance of that item, it will enter the second phase of Delphi. The item is deemed important if, in both the first phase and second phases of Delphi, > 75% of experts agree with its importance. To reduce the error, Wilcoxon and Bonferroni correction tests were used in addition to the experts blindly evaluating the information.

#### MDS content validity evaluation

After the Delphi phase, important items were extracted, and those with little importance were excluded. In this way, the initial MDS file was created and handed to the panel of experts participating in the Delphi phase to check its content validity in the following steps:

##### Content validity index (CVI)

The Content Validity Index (CVI) shows whether each MDS item is relevant to the main purpose of the study. The CVI must be measured for each item. Therefore, the initial MDS was emailed to the panel of experts, and the respondents were requested to rate each item on a four-point Likert scale. On this scale, a score of 1 indicates irrelevance and a score of 4 indicates the highest level of relevance. The experts had 10 days to return their responses. To calculate the CVI, the number of experts who gave the item a score of 3 or 4 was divided by the total number of experts. The acceptable value for CVI is 0.78%. Some chance is involved in calculating CVI. To eliminate this chance, we also measured S-CVI (universal agreement) and (average). It is recommended that the minimum S-CVI should be 0.8 to reflect content validity.

##### Calculation of kappa

Since there is some chance in calculating the CVI, another way to eliminate this chance is to use measure kappa for each item. The formula K = (I-CVI-PC) / (1-PC) was used to calculate kappa. The description of the kappa statistic for each item is as follows: Kappa > 0.74 means that the item is excellent for the MDS form; kappa of 0.6–0.74 means good, kappa of 0.4–0.59 means a moderate item that should be removed from the final MDS form.

##### Calculating content validity ratio (CVR)

After calculating the CVI, we measured the CVR for each item; after determining the importance and relevance of each item, we also measured the necessity of that item. To measure the CVR, we sent the filtered MDS from the previous steps to the panel of experts. The experts were asked to rate each item on a Likert scale of 1–3. On this scale, a score of 1 indicates the non-necessity of that item, and a score of 3 indicates its necessity. CVR was calculated using the formula CVR = (Ne—N / 2) / (N / 2). The experts had seven days to score the items.

##### Face validity check

This criterion evaluates the appearance of the final MDS file and whether such a tool is suitable for users. To calculate the face validity for each item, we sent the filtered MDS from the previous steps to the panel of experts. We evaluated each item in five domains: the technical content is fine, the continuity of items is fine, the language is understandable, terminology, and the given options are easy to understand. The panel of experts was asked to rate each item of MDS on a Likert scale of 1–4 in terms of the mentioned aspects. The formula Impact Score = Frequency (ratio of raters who scored 3 & 4) * Importance (mean score for the importance on the basis of domains) was used. The impact score for each item must be > 1.5; otherwise, it will be removed.

#### Design and construction of the suicidal behaviors surveillance system

**Fig. 2:**
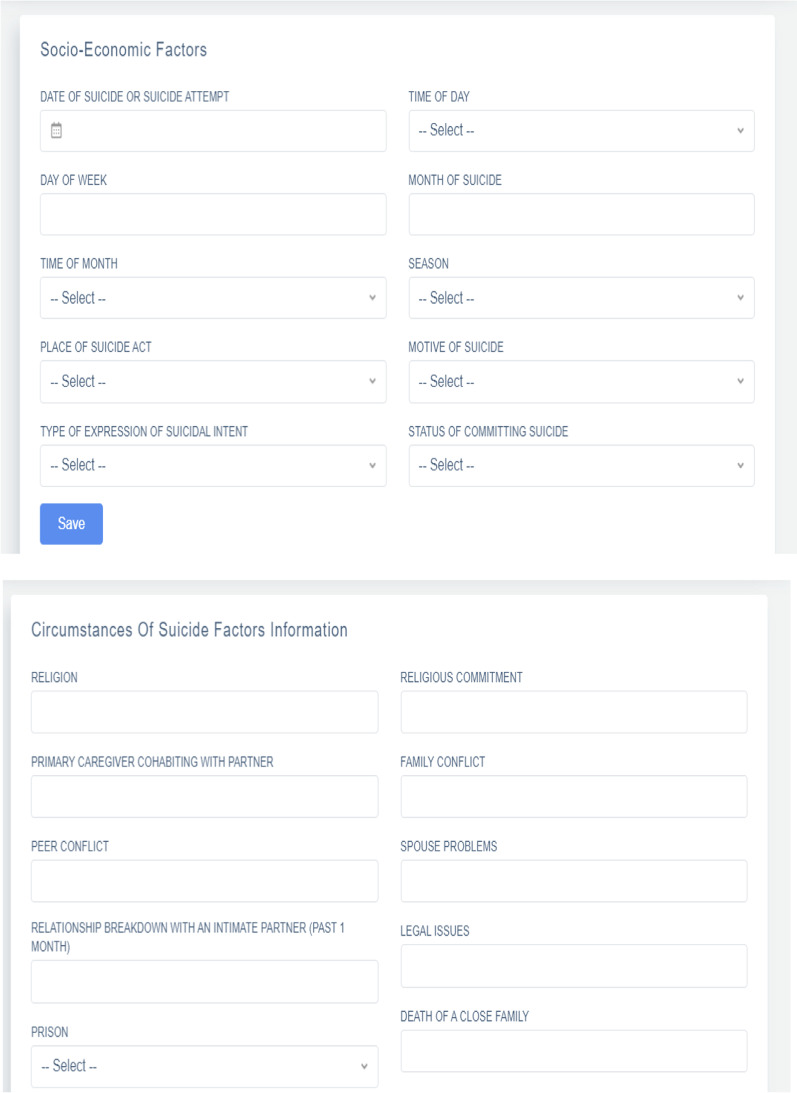
The user interface of the suicide surveillance system

A web-based suicide surveillance system was designed using Visual Studio 2019. This platform was used owing to its numerous benefits (e.g., cost-effective, scalable, accessible, user-friendly, fast, convenient, custom searchability, improved Intellicode, having a clipboard, and refactoring attributes) [26]. The developed system was implemented with the cascading style sheets (CSS) technology as a web-based program. CSS, along with the hypertext markup language (HTML), was used to describe the presentation of documents and set the document syntax, layout, display format, and visual effects (e.g., font type, color, spacing, and size). The code was written in JavaScript to design the website. Finally, the structured query language (SQL) was employed to develop the relational database (RDB). SQL provides efficient and systematic storage of data with high performance, availability, scalability, flexibility, management, and security [27] (Fig. 2).

#### Piloting the designed system

In this phase, a pilot study was conducted to assess the satisfaction level of the designed system from the users' point of view. For this end, 150 individuals who worked at suicide-related organizations such as healthcare settings and forensic medicine participated in this phase. The panel was selected based on the five inclusion criteria: (1) Familiarity with the evaluation of surveillance systems; (2) Awareness of the strengths and limitations of surveillance systems (3) Familiarity in recording the information of suicidal people, and organizational rules and principles of suicide data collection, (4) Having at least two years of work experience, and (5) Willingness to participate.

After selecting the panel, a consent form was sent to each participant. The purpose of the study was fully described. finally, a username and password were defined for each participant and the system link with the electronic questionnaire was emailed to them. In our study, the questionnaire designed in Mitchell et al. study [28], was used to observe the performance of the developed system. The questionnaire evaluates the suicidal behaviors surveillance system from three aspects. These aspects and their subsets include: 1) operational characteristics, including purpose and objective, data collection process, timelines, uniform classification systems, quality control measures systems security, confidentially, and privacy, 2) practical characteristics including data accessibility, usefulness, data analysis, guidance material to aid data interpretation, and 3) data quality characteristics including data completeness, sensitivity, specificity, positive predictive value, representative-ness.

The users’ panel rated all characteristics to evaluate either the data quality, operation, or practical capabilities of the suicidal surveillance system using a 7-point Likert scale from 'not at all' to 'extremely'. For a characteristic to be 'satisfaction' it was required to be judged by the majority of the panel as so, with a mean rating of 6.0 or higher. Finally, central and dispersion indicators of the panel's ratings were measured. Also, the degree of consensus on each aspect of the developed system was evaluated.

### Ethical considerations

The director of the research facility of the university approved the research protocol (approval ID: IR.ABADANUMS.REC.1400.122). All the respondents were required to sign a confidentiality agreement and study participation consent form before joining the expert panel. They were aware of the objectives of the study and were informed that their participation was voluntary and they were free to withdraw from the study at any time.

## Results

### Identification of potential data elements of suicidal behaviors surveillance

This step was performed under the supervision of an epidemiologist. By searching the online databases after removing the duplicates, 2236 articles were extracted in this stage. Then, the abstracts of the articles were screened which led to 154 articles, of which 100 articles were removed because did not address the suicide factors. In the final stage, the full text of 54 studies was assessed to extract the suicidal factors, and 20 articles were excluded for various reasons such as similarity (Fig. 1).

### Delphi survey to identify important MDS items

All the factors related to suicide and suicide attempt were extracted and a pool of data was formed. After preparing the initial pool of data, similar data were removed. The remaining factors were divided into different categories in the form of an electronic checklist. The participants could vote for each item in different stages and based on the purpose of the study. The questionnaire used in this stage had 250 items, and each item was related to suicide and suicide attempts. The panel of experts in the Delphi phase comprised 50 people, including psychiatrists, psychotherapists, social workers, epidemiologists, and staff at suicide statistics centers in health and police centers. Table 2 lists the characteristics of the participants in the Delphi phase.

**Table 2:** Characteristics of the participants in the Delphi phase

Variables	Frequency	Percentage
*Gender*		
Female	23	46
Male	27	54
*Educational*		
Psychologist	12	24
Psychotherapist	14	28
Epidemiologist	10	20
Social worker	14	28
*Age*		
30 – 40	15	30
40 – 50	27	54
> 50	8	16
*Work experience*		
< 10	10	20
10–15	15	30
15–20	18	36
20–25	4	8
> 25	3	6
Total	50	100

	Mean	SD
Age	36.4	± 6.4
Work experience	12.32	± 5.2

A 250-item checklist, developed using the suicide and suicide-related factors, was sent to the participants. In the first round of Delphi, 60 items were removed and 70 items entered the second round. In the second round of Delphi, 60 items were removed and 10 items were accepted. Finally, from the 250 items, 120 items were removed, and 130 items were considered important suicidal behaviors factors by experts and included in the MDS platform. In the Delphi phase, the Wilcoxon test and Bonferroni correction were performed to reduce type I error and ensure the accuracy of the answer (Table 3).

**Table 3:** Calculate CVI and Delphi phase for the administrative class

Admission class									
Items	Delphi phase						*Calculation of I-CVI*		Final decision
	Round 1			Round 2					
	Agree N (%)	Dis agree N (%)	Unsure N (%)	Agree N (%)	Dis agree N (%)	Unsure N (%)	Relevant (Rating 3 or 4)	I-CVIs	
Record number	100	0	0				50	1	Kept
Gender	100	0	0				50	1	Kept
Age	100	0	0				50	1	Kept
Birth date	96%	2%	5%				48	0.96	Kept
Marital status	88%	10%	2%				48	0.96	kept
Occupation/Job	92%	6%	2%				50	1	Kept
Employment status	98%	0	2%				50	1	Kept
Date of suicide	76%	22%	2%	80%	18%	2%	45	0.9	Kept
Suicide method	100%	0	0				50	1	Kept
Residence	92%	2%	6%				48	0.96	Kept
Home address	82%	18%	0				50	1	Kept
Education level	92%	0	8%				49	0.98	Kept
Racial status	86%	12%	2%				46	0.92	Kept
Healthcare setting name	90%	8%	2%				50	1	Kept
Visit type	78%	18%	4%	82%	18%	0	45	0.9	Kept
Subsequent consultation visits	92%	6%	2%				48	0.96	Kept
Ward admission	74%	22%	4%	78%	20%	2%	28	0.56	Removed
Physician admission	68%	30%	2%	74%	20%	6%	24	0.48	Removed
Referral institute	60%	30%	10%	76%	24%	0	23	0.46	Removed

### MDS content validity evaluation

After identifying the initial data items in the Delphi phase, 130 items were sent to a panel of 50 experts to calculate the CVI of each item and the relevance of each domain. At this stage, out of the initial 130 items, only 125 items were identified as relevant to the purpose of the study. Table 3 shows the CVI of the administrative class items. The S-CVI was measured to reduce the chances of experts agreeing with each item and the degree of relevance of each item to the objective of the study (Table 4).

**Table 4:** Calculate S-CVI for the administrative class

Ratings on a 119-Item of Admission class by 50 Experts: ItemsRated3or4ona4-Point Relevance Scale				
Items of administrative class of suicide MDS	The number giving a rating of 3 or 4 to the relevancy of item	I-CVIs	S-CVI/UA The proportion of items on a scale that achieves a relevance rating of 3 or 4 by all the experts	S-CVI/Ave Average of the I-CVIs for all items on the scale
Record number	50	1	S-CVI: 0.84 S-CVI/UA: 0.44	S-CVI/Ave: 0.893
Gender	50	1		
Age	50	1		
Birth date	48	0.96		
marital status	48	0.96		
Occupation/Job	50	1		
Employment status	50	1		
Date of suicide	45	0.9		
Suicide method	50	1		
Residence	48	0.96		
Home address	50	1		
Education level	49	0.98		
Racial status	46	0.92		
Healthcare setting name	50	1		
Visit type	45	0.9		
Visits followed by	48	0.96		

### CVR and modified kappa

In addition to calculating the S-CVI, the CVR and kappa of the items were calculated to eliminate the odds in two phases. At this stage, 125 items filtered from the previous phase were sent to the panel of experts after calculating the scores to compute the CVR and kappa. CVR and kappa were calculated for 125 items, of which 118 items were accepted and 10 items were deleted. Table 5 presents the CVR and kappa of each item in the MDS administrative class.

**Table 5:** Calculate content validity ratio, modified Kappa and face validity

content validity ratio, modified Kappa and face validity						
Items of administrative class of suicide MDS	The number giving a rating of 3 or 4 to the relevancy of item	CVR^a^	Pc^b^	K^c^	Face validity^d^	Interpretation
Record number	50	1	0/009 × ${10}^{-48}$	1	3.5	Excellent
Gender	50	1	0/009 × ${10}^{-48}$	1	3.5	Excellent
Age	50	1	0/009 × ${10}^{-48}$	1	3.5	Excellent
Birth date	48	0.92	0/01 × ${10}^{-45}$	0.96	2.86	Excellent
Marital status	48	0.96	0/01 × ${10}^{-45}$	0.96	2.86	Excellent
Occupation/Job	50	1	0/048 × ${10}^{-45}$	1	3.5	Excellent
Employment status	50	1	0/048 × ${10}^{-45}$	1	3.5	Excellent
Admission date	45	0.8	0/02 × ${10}^{-45}$	0.9	2.75	Excellent
Suicide method	50	1	4 × ${10}^{-45}$	1	3.5	Excellent
Residence	48	0.92	0/009 × ${10}^{-48}$	0.96	2.86	Excellent
Home address	50	1	0/01 × ${10}^{-45}$	1	3.5	Excellent
Education level	49	0.96	0/01 × ${10}^{-45}$	0.98	2.9	Excellent
Racial status	46	0.84	0/048 × ${10}^{-45}$	0.92	2.63	Excellent
Healthcare setting name	50	1	0/02 × ${10}^{-45}$	1	3.5	Excellent
Visit type	45	0.8	0/02 × ${10}^{-45}$	0.9	2.36	Excellent
Visits followed by	48	0.92	0/01 × ${10}^{-45}$	0.96	2.86	Excellent

^**a**^The formula of content validity ratio is CVR = (Ne–N/2)/ (N/2). In which the Ne is the number of panelists indicating "essential" and N is the total number of panelists. The numeric value of content validity ratio is determined by Lawshe Table. if CVR is bigger than 0.49, the item in the instrument with an acceptable level of significance will be accepted

^b^Pc (probability of a chance occurrence) was computed using the formula: pc = [N! /A! (N -A)!] *.5Nwhere N = number of experts and A = number of panelists who agree that the item is relevant

^**c**^K (Modified Kappa) was computed using the formula: K = (I-CVI- PC)/ (1- PC). Interpretation criteria for Kappa, using guidelines described in Cicchetti and Sparrow (1981): Fair = K of 0.40 to 0.59; Good = K of 0.60 to 0.74; and Excellent = K > 0.7

^**d**^For calculation, the formula Impact Score = Frequency (ratio of raters who scored 3 & 4) * Importance (mean score for the importance on the basis of domains) was used. The Impact Score for each item must be above 1.5 or it will be removed

### Face validity

Face validity was measured for each item of the final MDS validation. At this stage, the MDS was sent to the panel of experts. After measuring face validity, five items were deleted. Table 5 presents the face validity of each item of the administrative class. After completing all the steps, a total of 108 items remained in the final suicide MDS.

### Confirmed MDS

The final MDS platform of our study contained 108 items. We divided this dataset into eight main categories of administrative class, social factors, economic factors, environmental factors, clinical or psychopathological factors, and behavioral factors, circumstances of suicide factors, and outcome and follow-up. The final data elements of the MDS were grouped as follows:

1. Administrative class includes 15 data elements such as record number, sex, age, birth date, marital status, employment status, date of suicide, suicide method, residence, home address, education level, race, healthcare setting name, visit type, subsequent consultation visits.
2. Social class includes 28 data elements such as the number of household cohabitants, religion, religious commitment, primary caregiver cohabiting with a partner, family conflict, peer conflict, spouse problems, relationship breakdown with an intimate partner (past 1 month), legal issues, prison, death of a close family member, parental supervision, parents' separation, class social, living alone, abuse, lifetime abuse, position in the household, family structure, family size, social and teamwork activities, source of support and assistance, antisocial activities, marital-partner relationship difficulties, problems with familial relationships, acculturation, certain attitudes.
3. Environmental class includes five data elements: the place of suicide act, neighborhood environment, and place in the household, immigration, drudgery, and hazardous work.
4. Economic factors include four data elements that cause suicidal thoughts in the person: income status, work problems, level of socioeconomic welfare, and recent job loss.
5. Clinical or psychopathology factors include 24 data elements: the source of history-taking, current illness, suicidal ideation, history of suicide attempt, intensity of suicidal ideation, history of chronic diseases, chronic diseases (if yes), serious physical illness, serious physical illness (if yes, specify), felt depressed, drug history, a stressful life (stressful events), type of stressful event (if yes), lifetime psychotic events, melancholic features, lack of confidantes, self-harm (past year), family history of suicide attempt, mental illness/suicide in the family, history of mental illness, habitual poor coping, sleep disorder, unsatisfied with life, guilt.
6. Behavioral factors include 24 data elements about behavioral factors that predispose one to suicide and include sexual orientation, history of forced sexual intercourse, bullying victimization, substance dependence, cigarette smoking, alcohol consumption, suicidal ideation, suicide attempts, ongoing interpersonal conflict, domestic violence, confusion about duty, poor camaraderie at work.
7. Circumstances of suicide factors include four data elements: suicide method, the motive for suicide, type of expression of suicidal intent, and status of committing suicide.
8. Outcome and follow-up factors include six data elements related to patient outcome; if the outcome is alive: has complications, admission ward, discharge date, doing follow-ups (OPD), and type of follow-up.

### Piloting the suicidal behaviors surveillance system

**Fig. 3:**
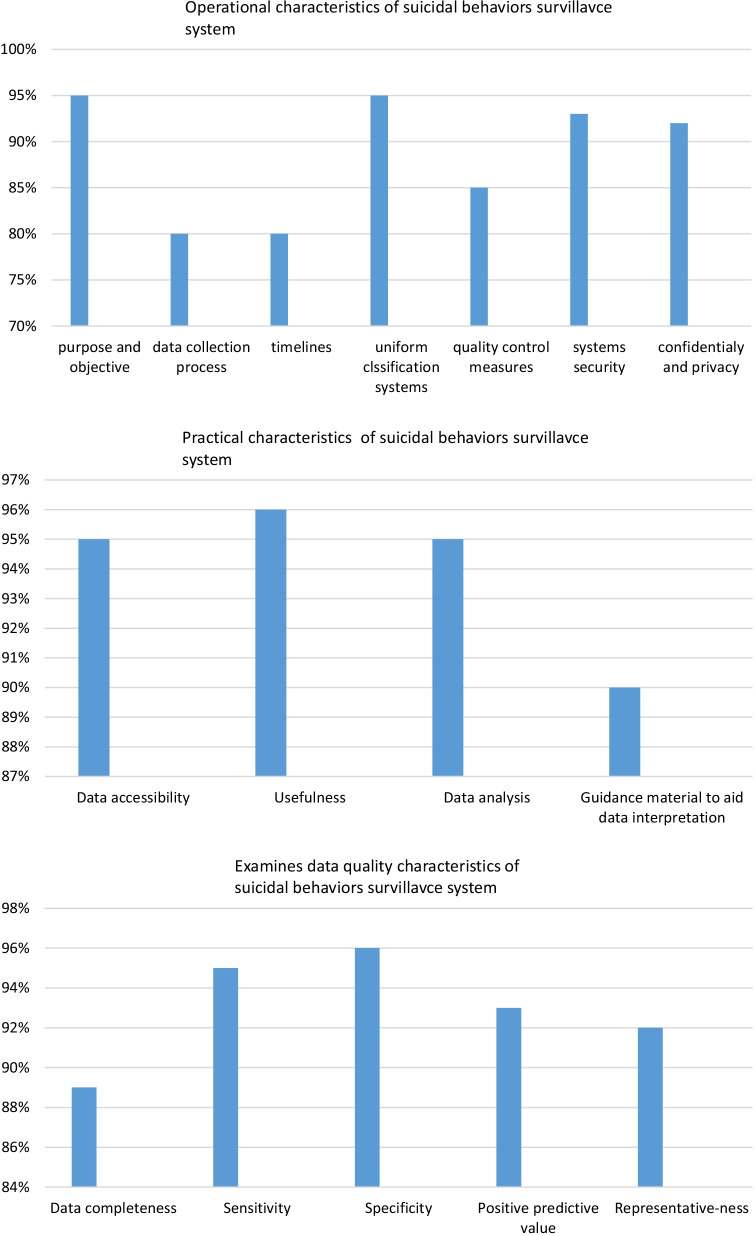
Data quality characteristics, Operational characteristics, and Practical characteristics of suicidal behaviors surveillance system

After developing the suicidal behaviors surveillance system, a survey was conducted on 150 participants to examine its data quality, operational, and practical characteristics. Table 6 shows the characteristics of the participants in this survey. A mean score of 6.0 or higher is adopted as a general cut-off to indicate a reasonably high level of satisfaction. Table 7 shows the participants' satisfaction scores from different aspects of the system. The mean satisfaction total rates (rate 6 to 7) were obtained for data quality, operational, and practical characteristics of 93%, 88.58%, and 94%, respectively. Figure 3 shows the percentage of each aspect of the system. Table 7 showed users’ panel ratings of the satisfaction of each characteristic to assess either the data quality, operation, or practical ability of the suicidal behaviors surveillance system.

**Table 6:** Characteristics of the participants in the survey

Variables	Frequency	percentage
Gender		
female	110	73.34
male	40	26.66

	Mean	SD
Age	36.4	± 6.4
Work experience	12.32	± 5.2

**Table 7:** Users panel rating of the satisfaction of each characteristic to assess either the data quality, operation or practical ability of the suicide behavioral surveillance system

Characteristic	Mean^a^	Median^b^	Standard Deviation	Range Consensus^c^	Rate number between (6–7) Percentage (%)
*Examines data quality characteristics of suicidal behaviors surveillance system*					
Data completeness	6.2	6	1	moderate	89%
Sensitivity	6.7	7	0.5	High	95%
Specificity	6.8	7	0.3	High	96%
Positive predictive value	6.4	7	0.7	High	93%
Representative-ness	6.3	7	0.8	High	92%
Mean Total (Rate 6–7) Percentage (%)	93%				
*Operational characteristics of suicidal behaviors surveillance system*					
Purpose and objective	6.7	7	0.5	High	95%
Data collection process	5.9	6	1.4	moderate	80%
Timelines	5.9	6	1.4	moderate	80%
Uniform classification systems	6.7	7	0.5	High	95%
Quality control measures	6	6	1.2	moderate	85%
Systems security	6.4	7	0.7	High	93%
Confidentially and privacy	6.3	7	0.8	High	92%
Mean Total (Rate 6–7) Percentage (%)	88.58%				
*Practical characteristics of suicidal behaviors surveillance system*					
Data accessibility	6.7	7	0.5	High	95%
Usefulness	6.8	7	0.3	High	96%
Data analysis	6.7	7	0.5	High	95%
Guidance material to aid data interpretation	6.2	7	0.9	High	90%
Mean Total (Rate 6–7) Percentage (%)	94%				

^a^Mean rating score using seven-point Likert scale (7 represents extremely satisfaction)

^b^Median rating score using seven-point Likert scale (7 represents extremely satisfaction)

^c^High consensus was considered to be 1 SD away from the mean, moderate consensus between 1 and 2 SDs away from the mean, and low consensus between 2 and 3 SDs away from the mean

## Discussion

To the best of our knowledge, this is the first attempt to develop a framework for suicidal behaviors surveillance in Iran's healthcare system. Suicide, suicide attempts, and suicide ideations are serious public health concerns demanding careful consideration. Suicide is a significant public health problem that has received substantial attention in Iran. Recently, academics and researchers have been gathering data on suicide and suicidal behaviors; however, there are variations about what data elements should be collected. Moreover, data about these conditions are inadequately collected and scattered amid different information systems across various organizations. Thus, there is a dire need for evidence-based accord on templates to establish a suicidal behaviors surveillance system for improving suicide monitoring.

Although there is sufficient research on suicide in Iran, no study has been found on developing a surveillance system for suicidal behaviors. Only the WHO framework to establish suicide surveillance was identified through the literature search. Therefore, this study aimed to design and develop a surveillance system for suicidal behaviors from an information management perspective to integrate community, clinical, and police data. This system can help lessen the under-reporting of suicide events due to sociocultural stigma in the context of Iran, and reduce the under-reporting of suicidal behaviors as compared to reliance on a single dataset from hospitals and police departments. This system may be appropriate for other low- and middle-income countries (LMIC) with insufficient official reporting and recording systems for suicidal behaviors.

In line with the literature, this study provides a data collection template that not only contains the epidemiological pattern of suicide but more significantly offers acceptable data set to support the design of suicide prevention programs, a module that is absent in most data collection tools in Iran. This feature can be provided in the system by considering data elements in the MDS which help capture required information regarding suicidal behaviors' risk factors.

Suicide prevention is a global health priority. It requires inclusive and multisectoral methods to address the risk at the individual, relationship, community, and social levels [29]. It is widely believed that suicide can be mitigated or prevented with the help of well-established suicide prevention plans. To prevent suicide and monitor suicidal behaviors, an effective and responsive surveillance system is critical. This system can enable multisectoral collaboration between various parties, including vital registrars, medical examiners, coroners, general practitioners (GPs), physicians, toxicology laboratories, hospitals, nursing homes, hospices, police departments, and policy-makers for timely control and prevention measures [30].

The efficiency of a suicide surveillance system depends on clinical data and reports from varied distributed sources, including police departments and medicolegal and clinical systems as data input. As such, the successful implementation of a suicidal behaviors surveillance system demands rich and comprehensible sets of data as the infrastructure for implementing electronic health and P4-medicine (Predictive, Preventive, Personalized, and Participatory) [31]. This system facilitates the management of suicide-related data for devising a better plan in the healthcare system and guiding research efforts on suicide.

The national surveillance of suicidal behaviors and outcomes can be challenging for several reasons. First, there is disagreement on what needs to be controlled [32]. Should we monitor all self-inflicted violence, complete suicides, suicide attempts, non-suicide self-harm, suicidal opinions, or a mixture of these? Second, there is no consensus about information systems applied to estimate the trend. In the existing information systems, data elements specific to suicide are often restricted, and the data gathered rarely provide sufficient information for identifying broad groups of high-risk populations, which can inform timely and reliable prevention and intervention actions [33, 34]. Third, suicide data can be unreliable for some reasons, including geographical variances in death examination approaches; when there are misclassifications in the outcome (suicides identified as non-suicides), e.g., suicides related to opioids are often misclassified as undetermined or accidental deaths; lack of financial support for coroners' or medicolegal offices to perform inclusive investigations on all appropriate occurrences; and variances in the degree to which potential suicides are examined to correctly determine the cause of death. Finally, there is a remarkable lag time in reporting suicide at the national level due to the complexity of the death certification and recording process. Suicide examination and reporting are complex processes that often involve various parties. Data from these parties must be accumulated at a national level in a unified and consistent manner. Nevertheless, due to the number of stages and complex courses involved, it is difficult to implement timely regulations for suicide prevention attempts or redirection of preventive resources [30, 32, 35].

Currently, surveillance systems are one of the most important public health needs. These systems can collect and analyze public health threats data, including suicide, and support the decision-making process [36]. The system designed in our study also has this advantage. It can be used in planning, budgeting, and evaluating intervention measures, decision-making, and research studies. Another salient feature of the developed system is that it identifies subgroups vulnerable to suicide and can guide proper health planning for them. This feature has been expressed in some studies [37, 38].

A guideline provided by the WHO was adopted to develop the suicide surveillance system in our study [39]. With respect to the country's need for a suicide surveillance system, this system can be used as a reference for reporting to the WHO. A major challenge in Iran is that suicide data are recorded in different organizations, and there is no appropriate coordination between them for planning and preventive policies [16]. We hope that the suicidal behaviors surveillance system developed in our study can coordinate suicide data collection among various organizations in the country, which is in line with the policy of the WHO. A basic prerequisite for designing an information system is the precise determination of its data elements; in this study, we first determined the required data elements of suicide.

In Iran, following the statement of the WHO, surveillance has been considered by health policies and, therefore, there is no obstacle to the design of the surveillance system. Moreover, the data of rural and native centers of each region are not recorded properly. Herein, we developed a framework that could be used in other centers, including forensics and the police department, to record information. On the other hand, this system should be usable in other areas, including rural and marginal areas, so the system was designed to be simple and easy to use. In Iran, as in some other countries, many suicidal deaths are not considered due to taboos; thus, this system can be implemented in other centers such as police and forensic medicine departments in addition to healthcare systems. In Iran, suicide taboos and the existence of legal problems have led to the refusal to report suicide, and this may impact the performance of this system. Still, the features of the developed surveillance system can lead to planning on laws related to suicide, and this system can help improve suicide reporting and prevention. Other features of the designed system lead to the exact cause of death and a more accurate expression of statistics related to mortality. On the other hand, the presence of epidemiological factors in this system will determine the risk factors of suicide, and this will be a key factor in determining the health policies of a government in the field of suicide.

In terms of technical infrastructure, this system was examined and tested in various organizations. We found that 93% of the participants were generally satisfied with the data quality characteristics, 88.58% with operational characteristics, and 94% with practical characteristics. These high levels show that the developed system can meet the needs and expectations of its users.

### Study strengths and limitations

Our study benefits from an evidence-based approach and experts’ collective wisdom in determining the suicidal behaviors' MDS by carrying out a comprehensive literature review and structured Delphi rounds. The experts in our study confirmed that the standardization of a data set is valuable for suicidal behaviors, as it provides the integration of data among involved organizations. We hope that our study can demonstrate the worth of calibration and integration of suicide data as a crucial step towards the implementation of suicide prevention and surveillance programs. Furthermore, it helps improve the coordination of scientific research and practices to successfully address suicide and suicidal behaviors. However, our method has some limitations that need to be addressed. First, given the unfamiliarity of many aspects of suicidal manners, further external validation is required; thus, conducting a pilot study with a more extensive literature review and a larger panel of experts could augment the MDS. Participation of a limited number of specialists from one province is another significant limitation of the study. Hence, the developed MDS must be evaluated from the standpoint of more multidisciplinary teams all over the country. Finally, the Delphi technique was used to reach an agreement on the suicide MDS. This technique has been proven to be appropriate for the analysis of the requirements of information systems [40], but most opinions might be marginalized.

### Implications for future studies

Due to the obsolescence of traditional and basic electronic information exchange methods (fax, e-mail, Outlook Express), the suicidal behaviors surveillance will require higher and more sophisticated levels of information exchange technologies. Designing structural and content standards for interoperability between information systems leads to the integration of distributed and islanded systems in different organizations. Thus, inter-organizational integration and shared information understanding become possible. The MDS developed in our study provides a scientific and evidence-based data collection tool for uniform collection of data on suicidal behaviors by various involved organizations. It can assist data integration in suicide-related information systems and contribute to interoperability in the context of suicide. It is recommended that upcoming research focus on the technical aspects of interoperability in this domain.

## Conclusions

This is the first effort to develop and evaluate a surveillance system for suicidal behaviors in Iran. By integrating data from clinical systems, medicolegal, and police records, the developed system can be an effective tool to collect more complete data on suicidal behaviors, identify geographical trends and methods of suicide, and understand the impact of suicide prevention programs. Therefore, the integration of various suicide-related systems is likely to address the problem of suicide and attempted suicide underreporting in Iran. This template should also be periodically revised to warrant harmony so that it remains consistent with current suicide.

## Data Availability

The datasets used and/or analyzed during the current study are available from the corresponding author on reasonable request.

## Abbreviations

MDS: Minimum data set
WHO: World health organization
SDLC: System development life cycle
CVI: Content validity index
CVR: Content validity ratio
CSS: Cascading style sheets
HTML: Hypertext markup language
SQL: Structured query language
RDB: Relational database
